# Acute pre‐exercise oral intake of cacao polyphenols attenuates central fatigue development during sustained low‐intensity ankle dorsiflexion exercise in healthy young male adults

**DOI:** 10.14814/phy2.71095

**Published:** 2026-09-07

**Authors:** Ikumi Morikawa, Ashiyu Ono, Yuta Osada, Yasuyuki Fujii, Midori Natsume, Naomi Osakabe, Ryota Akagi

**Affiliations:** ^1^ Graduate School of Engineering and Science Shibaura Institute of Technology Saitama‐shi Saitama Japan; ^2^ SIT Research Laboratories Shibaura Institute of Technology Saitama‐shi Saitama Japan; ^3^ Meiji Co., Ltd. Hachioji‐shi Tokyo Japan; ^4^ Meiji Holdings Co., Ltd. Hachioji‐shi Tokyo Japan; ^5^ Bioscience Course, Bioscience and Engineering Program, College of Systems Engineering and Science Shibaura Institute of Technology Saitama‐shi Saitama Japan; ^6^ Sports Engineering Course, Bioscience and Engineering Program, College of Systems Engineering and Science Shibaura Institute of Technology Saitama‐shi Saitama Japan

**Keywords:** maximal voluntary isometric contraction, neuromuscular fatigue, randomized crossover trial, salivary biochemical markers, voluntary activation

## Abstract

This double‐blind, randomized, crossover study investigated whether acute cacao polyphenol (CP) ingestion attenuates neuromuscular fatigue in 19 healthy young male adults. After consuming CP or placebo, participants performed sustained isometric ankle dorsiflexion at 25% of maximal voluntary isometric contraction (MVIC) torque until task failure. Neuromuscular function (MVIC torque, voluntary activation [VA%], doublet torque) and electromyographic activity were assessed before, immediately after, and during recovery (1 and 5 min). Salivary chromogranin A and α‐amylase were measured at baseline and 1 min post‐task. Only VA% and chromogranin A showed significant condition × time interactions (*p* = 0.027–0.039). VA% significantly decreased from before to immediately after the task and 1 min post‐task in both conditions (*p* ≤ 0.002), with significantly higher values in the CP condition than in placebo immediately after and 1 min post‐task (*p* ≤ 0.037). Chromogranin A significantly increased from baseline to 1 min post‐task in the placebo condition (*p* < 0.001) but not in the CP condition (*p* = 0.083). These findings suggest that acute CP ingestion attenuates the decline in central motor drive and autonomic stress responses following strenuous exercise, without altering performance or peripheral recovery, supporting its potential as a nutritional strategy for modulating central fatigue.

## 1 INTRODUCTION

Neuromuscular fatigue is a major factor limiting performance across various sporting activities and is characterized by reduced maximal voluntary force or power arising from interactions between central and peripheral mechanisms (Carroll et al., 2017). Peripheral fatigue reflects processes distal to the neuromuscular junction, whereas central fatigue originates within motoneurons and the central nervous system (Carroll et al., 2017). Although central fatigue may initially recover rapidly, complete recovery is often prolonged and varies with exercise intensity and duration (Carroll et al., 2017). Beyond performance decline, central fatigue is hypothesized to serve as a protective mechanism limiting disturbances in muscle homeostasis. Exercise‐induced metabolic alterations provide inhibitory feedback signals to the central nervous system via group III and IV muscle afferents, thereby preventing peripheral fatigue from exceeding a critical threshold (Hureau et al., 2018). This aligns with the muscular wisdom hypothesis, which proposes that reduced motor unit firing rates preserve electrical propagation (Zero & Rice, 2025), and with the sensory tolerance limit concept, whereby the central nervous system integrates feedback and feedforward signals to maintain activity within tolerable limits (Hureau et al., 2018). Because this central inhibitory process ultimately determines task failure (Hureau et al., 2018; Zero & Rice, 2025), strategies that mitigate declines in voluntary neural drive and enhance recovery may extend performance more effectively than those targeting peripheral factors alone.

Central fatigue is strongly influenced by exercise‐induced changes in cerebral monoamines, with an elevated serotonin/catecholamine ratio promoting fatigue (Meeusen et al., 2006). Although caffeine counteracts this by antagonizing adenosine receptors and facilitating dopaminergic activity (Solinas et al., 2002), its use may be limited by safety concerns. Recommended intake limits aim to minimize acute effects such as palpitations and insomnia, as well as potential long‐term risks (de Souza et al., 2022). Accordingly, safe, natural alternatives that modulate monoaminergic activity and attenuate central fatigue are needed.

Polyphenols have emerged as promising candidates. These structurally diverse plant metabolites are abundant in plant‐based foods and have an established safety profile (Silva & Pogačnik, 2020). Cacao polyphenols (CP) primarily comprise flavanols, including catechins, their polymers, and procyanidins (Wollgast & Anklam, 2000). Chronic CP intake has been shown to enhance hippocampal‐dependent memory, particularly in individuals with lower diet quality (Brickman et al., 2023). Acute CP ingestion also improves mental tracking (Massee et al., 2015), mood (Pandolfi et al., 2016), and attention (Gratton et al., 2020), and transiently increases cerebral blood flow. Our recent study (Fujii et al., 2025) supports these findings and showed that oral CP administration in mice induced arousal and enhanced short‐term memory despite low bioavailability. These effects likely involve gastrointestinal stimulation that activates the locus coeruleus–noradrenergic network, increasing noradrenaline in the hypothalamus and brainstem and elevating neuroendocrine stress responses. Collectively, these findings suggest that CP may potentially preserve neural drive and alleviate central fatigue by modulating autonomic and monoaminergic systems via the gut–brain axis; sensory properties such as astringency may contribute to these effects. However, whether these acute responses are unique to CP or represent broader dietary polyphenol effects, and whether these microvascular or monoaminergic mechanisms operate directly during fatiguing motor tasks in humans, remain to be established.

Among single‐joint tasks, ankle dorsiflexion provides a simple and reliable model for assessing neuromuscular function. The tibialis anterior (TA) constitutes more than half of dorsiflexor muscle volume (Nakagawa et al., 2005) and generates most dorsiflexion torque (Fukunaga et al., 1996). It also exhibits high voluntary activation, often approaching 100% in a non‐fatigued state (Akagi et al., 2020), making it sensitive to changes in central drive. Because sustained low‐intensity contractions can induce central fatigue (Place et al., 2009), including in the ankle dorsiflexors (Lowe et al., 2023), we investigated whether a single oral dose of CP before sustained low‐intensity ankle dorsiflexion attenuates neuromuscular fatigue and enhances recovery. By integrating neuromuscular measures with salivary biomarkers, we aimed to determine whether CP preserves neural drive and mitigates central fatigue by modulating central nervous system responses to physiological stress. These findings may inform nutritional strategies for optimizing recovery in athletes, particularly when central fatigue predominates.

## 2 METHODS

### 2.1 Ethical approval

The study was conducted in accordance with the Declaration of Helsinki and was approved by the Ethical Review Committee of Shibaura Institute of Technology (no. 24‐029) after registration in UMIN‐CTR (UMIN000055775). Participants were informed of the study purpose and potential risks and provided written informed consent.

### 2.2 Sample size calculation and participants

We examined changes over time in skeletal muscle‐related variables between two conditions (CP and placebo) before (Pre), immediately after (Post), and at 1 and 5 min after a fatiguing task (R1 and R5). We performed an a priori power analysis for a two‐way repeated‐measures analysis of variance (ANOVA) to estimate the sample size for the subsequent linear mixed model (LMM) analysis owing to the difficulty of pre‐specifying correlation structures in LMMs. We conducted the power analysis using G*Power software (version 3.1.9.4; Kiel University, Kiel, Germany). The input parameters were as follows: statistical test = “ANOVA: Repeated measures, within factors”; effect size *f* = 0.25 (assuming a medium effect size [Cohen, 1988]); *α* error probability = 0.05; power (1 – *β* error probability) = 0.80; number of groups = 1; number of measurements = 8; correlation among repeated measurements = 0.50; and nonsphericity correction *ε* = 1. The calculated sample size was 16. To account for potential dropouts and avoid the confounding effects of the menstrual cycle on neuromuscular function (Ansdell et al., 2019) and monoaminergic responses (White et al., 2002), we limited recruitment to male participants and enrolled 19 healthy young male adults (mean ± standard deviation [SD]: age, 22 ± 2 years; height, 173.0 ± 6.2 cm; body mass, 60.4 ± 9.2 kg). Participants were recruited through direct verbal invitations and individual emails. Participants were free of cardiovascular, neuromuscular, and orthopedic lower‐body conditions; had not smoked within the preceding 6 months; and maintained regular routines, with stable sleep (6–8 h/night) and consistent physical activity, as verified by questionnaire.

### 2.3 Experimental design

This double‐blind, randomized crossover study compared CP and placebo, with the conditions separated by a 1–2‐week washout period. A familiarization session was conducted ≥1 week before the first experimental session; participants practiced maximal voluntary isometric contraction (MVIC) torque measurement and a portion of the fatiguing task.

Participants maintained normal routines throughout the study. For 3 days before each session, they refrained from strenuous exercise and alcohol and maintained adequate sleep. They photographed their meals the day before each session to ensure dietary consistency. On experimental days, participants arrived after breakfast. Testing times were matched for each participant across sessions (11:30 a.m. or 1:30 p.m.) to minimize circadian effects. Upon arrival, they consumed a standardized lunch (Donbei Kitsune Udon; Nissin Food Products Co., Ltd., Tokyo, Japan) to control nutritional status. Thirty minutes later, they ingested CP or placebo capsules. To obtain baseline measurements unaffected by supplementation, as capsule dissolution and absorption require ~15 min, pre‐measurements began 5 min post‐ingestion and were completed within ~10 min, before absorption or sensory signaling commenced. The fatiguing task began 45 min post‐ingestion. The 45‐min interval was selected to coincide with capsule breakdown and the initiation of gastrointestinal/sensory signaling via the gut–brain axis (Fujii et al., 2025), rather than peak systemic metabolite concentrations.

**FIGURE 1:**
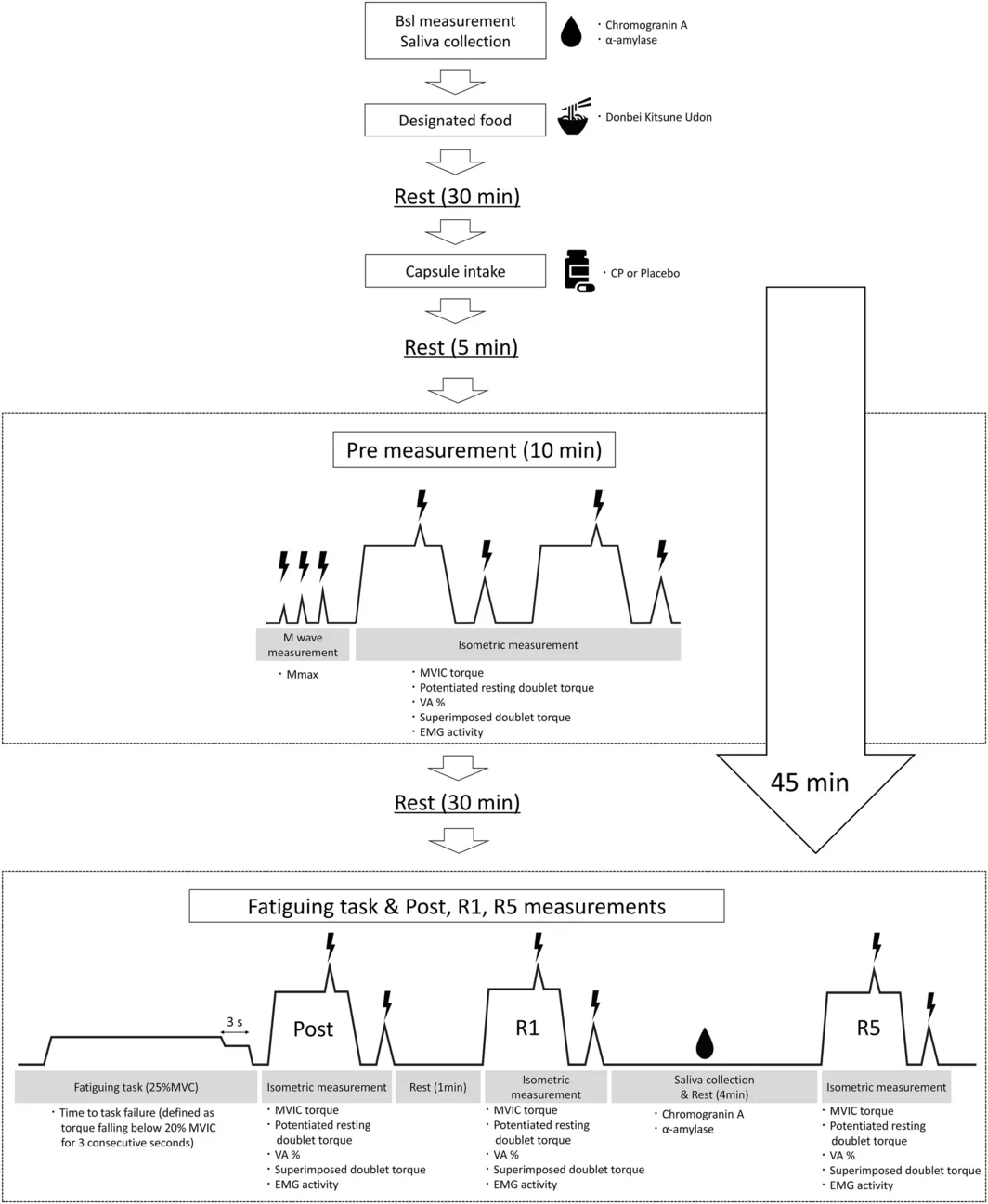
Overview of the experimental timeline. Bsl, before ingestion of the designated food product; CP, cacao polyphenols; EMG, electromyography; Mmax, peak‐to‐peak maximal compound muscle action potential amplitude; MVIC, maximal voluntary isometric contraction; Post, immediately after the fatiguing task; Pre, before the fatiguing task; R1, recovery at 1 min post‐task; R5, recovery at 5 min post‐task; VA%, voluntary activation.

For the right‐leg dorsiflexors, we measured the following variables at Pre: peak‐to‐peak maximal compound muscle action potential amplitude (Mmax), MVIC torque, voluntary activation (VA%), superimposed doublet torque (SDT), and potentiated resting doublet torque (PRDT). Participants then performed the fatiguing task. MVIC torque, VA%, SDT, and PRDT were reassessed at Post, R1, and R5. Surface electromyography (EMG) signals from the TA and soleus (SOL) were recorded during all MVICs to evaluate the neuromuscular activity of the agonist and antagonist muscles, respectively. Changes in MVIC torque indicated overall neuromuscular fatigue (central and peripheral components) (Akagi et al., 2020), whereas changes in PRDT indicated peripheral fatigue (Burnley et al., 2012). Central fatigue was assessed using changes in VA% (Burnley et al., 2012) and root mean square EMG (RMS‐EMG) normalized to Mmax (Place et al., 2010). We collected saliva samples for stress biomarker analysis at baseline (Bsl; pre‐ingestion) and R1. The measurement sequence is shown in Figure 1.

During measurements and the fatiguing task, participants lay supine on a dynamometer (CON‐TREX; PHYSIOMED, Schnaittach, Germany) with the right foot fixed at 30° plantar flexion using nonelastic straps. This ankle position lies within the plateau region of the torque–angle relationship for maximal dorsiflexion torque (Akagi et al., 2020) and was expected to enhance the signal resolution and sensitivity of the interpolated‐twitch technique. The ankle joint and dynamometer axes were aligned. Participant positioning was standardized across sessions using scale markers. We provided strong verbal encouragement during the MVICs and fatiguing tasks.

Torque and EMG signals were sampled at 2 kHz and digitized using a PowerLab 16/35 (ADInstruments, Bella Vista, NSW, Australia), and recorded using LabChart software (v8.1.5; ADInstruments). During the fatiguing task, real‐time torque was displayed on a monitor and goggles worn by the participant (NR‐7100RGL; XREAL, Beijing, China).

### 2.4 CP and placebo intervention

Active capsules contained Meiji Cacao Flavanol Powder™ (Meiji Co., Ltd., Hachioji‐shi, Tokyo, Japan). Placebo capsules contained dextrin with food‐grade theobromine at matched doses but no cacao flavanol powder. The placebo and CP capsules were prepared in the laboratory using 0.68‐mL Capsugel capsules (DR T & T Health UK Ltd., UK). Capsules were identical in taste and appearance. Only the manufacturer was aware of the capsule contents, thereby maintaining blinding. Formulation details are provided in Table 1. The administered dose of 500 mg CP was selected on the basis of previous acute human studies demonstrating that a single dose of cacao flavanols (~500 mg) improves cognitive control and acute cerebrovascular responsiveness and modulates autonomic activity (Gratton et al., 2020; Massee et al., 2015).

**TABLE 1:** Nutritional composition and polyphenol content of the cacao polyphenols and placebo capsules.

	Placebo (mg/pill)	Test food (mg/pill)
Polyphenol	N.D.	500
(+)‐catechin	N.D.	2.7
(−)‐epicatechin	N.D.	31.3
Procyanidin B2	N.D.	14.5
Procyanidin B5	N.D.	1.9
Procyanidin C1	N.D.	6.6
Cinnamtannin A2	N.D.	2.9
Xanthine derivatives		
Theobromine	180.8	180.8
Caffeine	N.D.	N.D.

*Note*: Polyphenols and xanthine derivatives were determined by high‐performance liquid chromatography. Total polyphenol content was determined by the Folin–Ciocalteu method.

Abbreviation: N.D., not detected.

### 2.5 EMG setup

Participants stood with their arms relaxed. The right lower‐leg length (popliteal crease to lateral malleolus) was measured to the nearest 0.5 cm. Measurement marks were placed at 30% and 60% of the proximal lower‐leg length. An ultrasound system (SUPERSONIC™ MACH 20, Hologic Inc., Marlborough, MA, USA) with a 38‐mm linear probe (10L2 Transducer, 10 MHz) was used to identify EMG recording sites for the TA and SOL. For each muscle, the mediolateral midpoint was marked. The skin at the electrode sites and over the left lateral malleolus (reference) was abraded and cleaned. Bipolar surface electrodes with preamplifiers (1 × 10 mm, 10‐mm interelectrode distance; DE‐2.1, Delsys, Inc., Natick, MA, USA) were placed over each site. EMG signals were bandpass filtered at 20–450 Hz (Bagnoli 8 EMG System, Delsys, Inc., Natick, MA, USA).

### 2.6 *M*max

Single‐twitch responses of the SOL and TA were determined using a constant‐current, variable‐voltage stimulator (DS7A, Digitimer Ltd., Hertfordshire, UK) and a controller (LabChart v8.1.5; ADInstruments, Bella Vista, NSW, Australia). The stimulation sites were identified using anatomical landmarks: the site to stimulate the deep fibular nerve (innervating the TA) was identified by palpating the region posterolateral to the fibular head and moving distally, whereas the site to stimulate the tibial nerve (innervating the SOL) was identified by palpating the area deep in the popliteal fossa. Once the sites were located, a custom‐built clinical bar electrode consisting of two 10‐mm‐diameter pellets with an interelectrode distance of 30 mm was coated with conductive gel and positioned over the respective nerve. Percutaneous stimulation was delivered as a 100‐μs rectangular pulse. For the SOL, the stimulus intensity was progressively increased from 50 mA in 50‐mA increments until the single‐twitch torque plateaued (150–750 mA). For the TA, the intensity was increased from 20 mA in 20‐mA increments until a plateau was reached (40–160 mA). Mmax was assessed for both muscles at these plateau intensities.

### 2.7 MVIC torque, VA%, SDT, PRDT, and EMG

Following a warm‐up of 2–3 submaximal contractions, peak torque during a 3‐s MVIC of the dorsiflexors was measured in two trials separated by 1‐min. If the difference exceeded 10% of the higher value, additional trials were performed until the difference between the highest and second‐highest values was <10%, with 1 min of rest between trials. Two supramaximal stimulations were delivered ~2 and 3 s after MVIC onset and after its completion, respectively, to determine SDT, PRDT, and VA% (= [1 − (SDT/PRDT)] × 100). The stimulation current was set to 140% (Akagi et al., 2020) of the TA twitch torque plateau intensity (56–224 mA), and stimulation was delivered as a 100‐Hz doublet. Root‐mean‐square EMG values (RMS‐EMG values) of the TA and SOL during MVIC were calculated over a 500‐ms window, from 550 to 50 ms before delivery of the superimposed doublet stimulation. The highest torque value was defined as MVIC torque; potentiated twitch torque, VA%, and RMS‐EMG from that trial were analyzed. RMS‐EMG values were normalized to Mmax of the respective muscles. Torque data were low‐pass filtered at 500 Hz, and MVIC torque, potentiated twitch torque, and VA% were analyzed offline.

**FIGURE 2:**
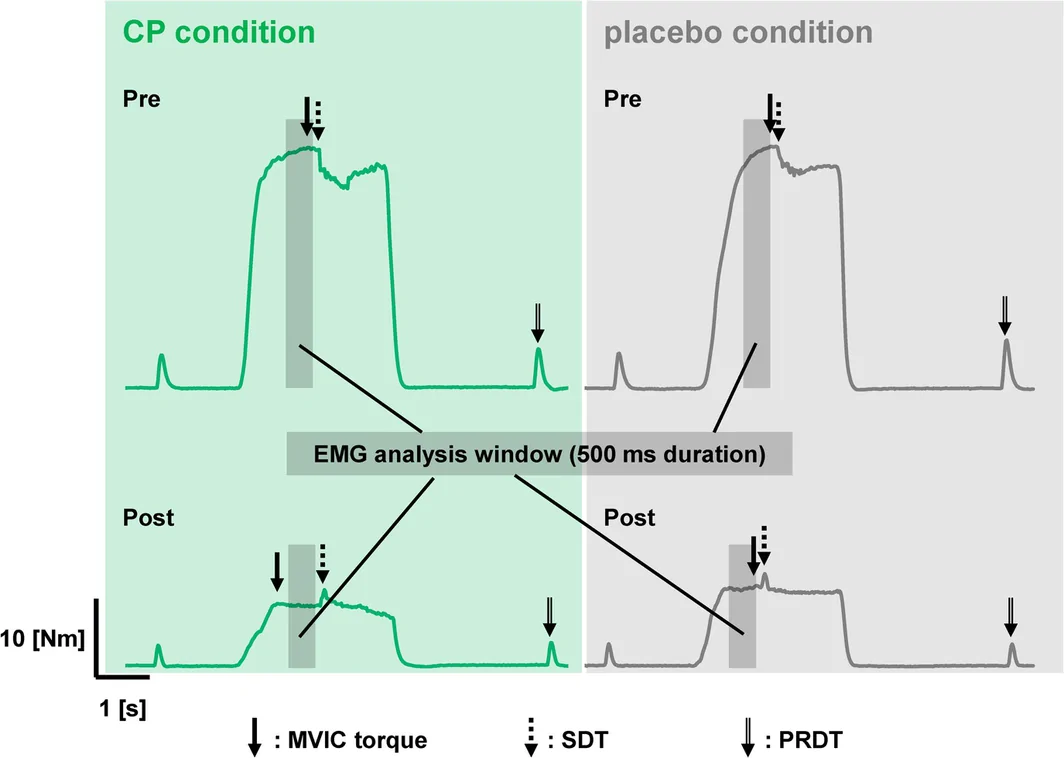
Representative torque traces during a maximal voluntary isometric contraction (MVIC) with the interpolated twitch technique. Representative torque traces are shown before (Pre) and immediately after (Post) the fatiguing task, illustrating the electromyography (EMG) analysis window as well as the torque responses to supramaximal doublet stimulations during (superimposed doublet torque, SDT) and after (potentiated resting doublet torque, PRDT) the MVIC for both the cacao polyphenols (CP) and placebo conditions.

At Post, R1, and R5, participants performed a single 3‐s dorsiflexor MVIC. VA%, SDT, PRDT, and RMS‐EMG values of the TA and SOL were assessed, with RMS‐EMG values normalized to the previously obtained Mmax values. Representative torque traces showing MVIC torque, SDT, and PRDT are presented in Figure 2.

### 2.8 Fatiguing task

Participants wore virtual reality goggles (NR‐7100RGL; XREAL, Beijing, China) displaying a mirrored view of the PC screen for real‐time torque monitoring. Two horizontal lines representing 25% and 20% of MVIC torque were displayed. Participants maintained torque at 25% MVIC; task failure was defined as torque falling below 20% MVIC for 3 consecutive seconds.

### 2.9 Salivary chromogranin A and α‐amylase

Saliva samples were collected using Salivette® (Sarstedt, Nümbrecht, Germany) to evaluate stress responses. Chromogranin A and α‐amylase served as markers of sympathetic activity (Chojnowska et al., 2021). Samples were centrifuged at 3000 rpm for 15 min, and the supernatant was stored at −80°C. Chromogranin A was quantified using a Human Chromogranin A ELISA Kit (YK070, Yanaihara Institute Inc., Shizuoka, Japan), and α‐amylase was quantified using a Salivary α‐amylase EIA Kit (1‐1902, Salimetrics, LLC, State College, PA, USA), in accordance with the manufacturers' instructions. Measurements were obtained using an Arvo ×4 microplate reader (PerkinElmer Inc., Waltham, MA, USA).

### 2.10 Statistical analyses

Data are presented in the text as mean ± SD. We conducted analyses using JASP (version 0.98.1; JASP Team, Amsterdam, The Netherlands) and Excel (2021; Microsoft Corp., Redmond, WA, USA). Statistical significance was set at *p <* 0.05. Because of a stimulator malfunction, one participant had incomplete data at one time point; thus, PRDT, VA%, SDT, and normalized RMS‐EMG values were analyzed using data from 18 participants.

Time to task failure was compared between conditions using a paired *t*‐test after normality was confirmed (Shapiro–Wilk test). For other variables, LMMs were used with condition, time, and the condition × time interaction as fixed effects, and participant ID as a random intercept (Type III sums of squares, Satterthwaite approximation for degrees of freedom). To account for between‐participant variability in responses, random slopes for condition, time, and their interaction were initially included in the model. However, when the inclusion of random slopes produced singular models (overparameterization), the random‐slope structure was simplified or removed to ensure model convergence and parsimony. For chromogranin A and α‐amylase, the model included condition (CP, placebo) and time (Bsl, R1). For MVIC torque, PRDT, VA%, SDT, and normalized RMS‐EMG values, the model included condition and time (Pre, Post, R1, R5). When significant interaction or time main effects were observed for variables with four time points, post hoc pairwise comparisons among all six pairs of time points (Pre vs. Post, Pre vs. R1, Pre vs. R5, Post vs. R1, Post vs. R5, and R1 vs. R5) were conducted using the Benjamini–Hochberg false discovery rate correction (within each condition for a significant interaction or using pooled data across conditions for a significant main effect of time).

## 3 RESULTS

Time to task failure was 515 ± 119 s in the CP condition and 519 ± 109 s in the placebo condition. The conditions did not differ significantly (*t*(18) = 0.170, *p* = 0.867).

**FIGURE 3:**
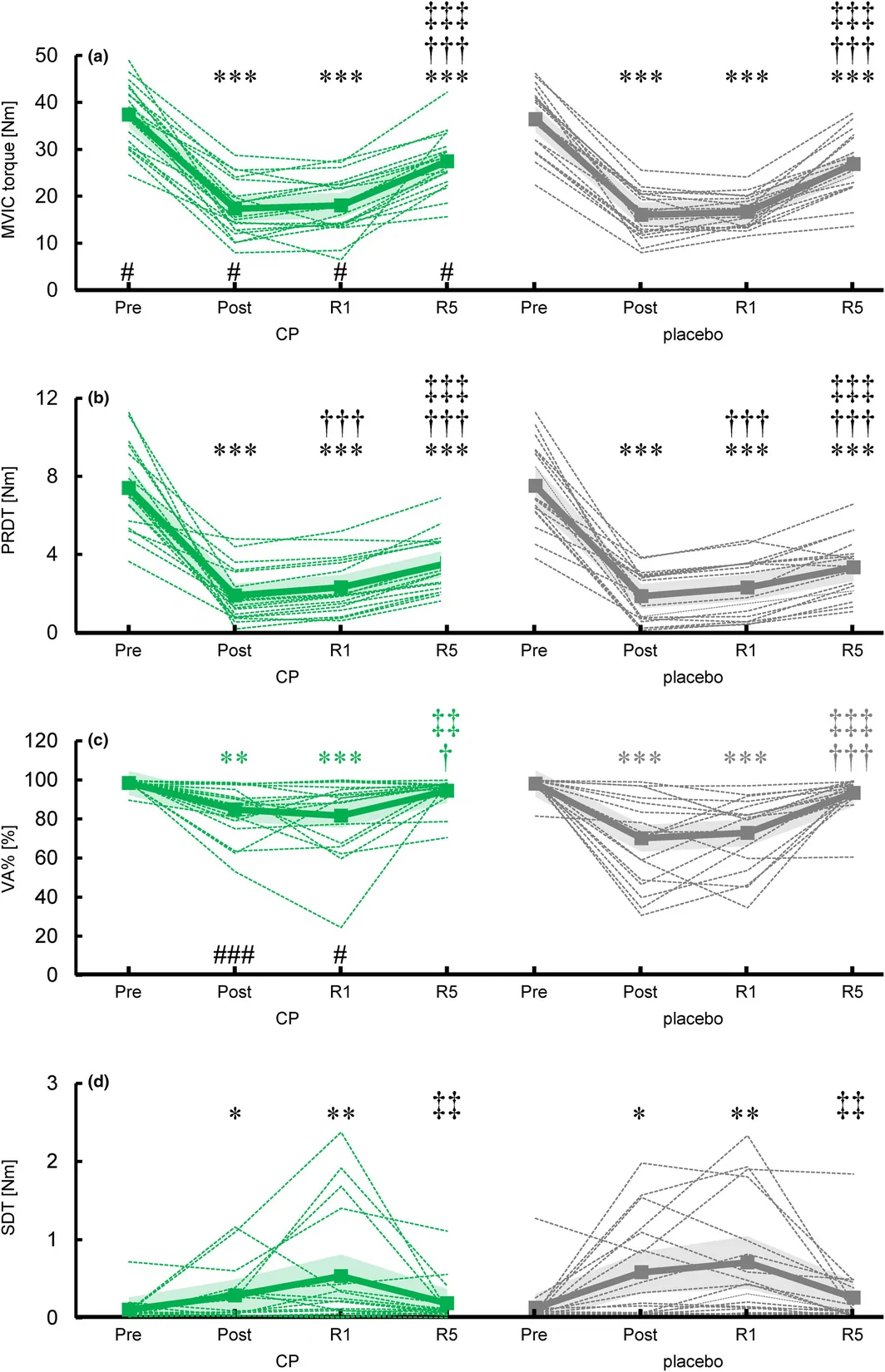
Time courses of maximal voluntary isometric contraction (MVIC) torque (*n* = 19; a), potentiated resting doublet torque (PRDT) (*n* = 18; b), voluntary activation (VA%) (*n* = 18; c), and superimposed doublet torque (SDT) (*n* = 18; d) of the dorsiflexors in the cacao polyphenols (CP; green) and placebo (gray) conditions. Data are presented as individual participant values (dashed lines) and estimated marginal means (solid lines with squares). Shaded bands represent the 95% confidence intervals derived from the linear mixed model. Measurements were taken at four time points: Before (Pre), immediately after (Post) the fatiguing task, and during recovery at 1 and 5 min post‐task (R1 and R5). Black * (*p* < 0.05; vs. Pre), ** (*p* < 0.01; vs. Pre), *** (*p* < 0.001; vs. Pre), ††† (*p* < 0.001; vs. Post), ‡‡ (*p* < 0.01; vs. R1), and ‡‡‡ (*p* < 0.001; vs. R1) symbols indicate significant differences across time points derived from a significant main effect of time with no significant condition × time interaction. Colored ** (*p* < 0.01; vs. Pre), *** (*p* < 0.001; vs. Pre), † (*p* < 0.05; vs. Post), ††† (*p* < 0.001; vs. Post), ‡‡ (*p* < 0.01; vs. R1), and ‡‡‡ (*p* < 0.001; vs. R1) symbols (green: CP; gray: Placebo) indicate significant differences between time points derived from post hoc pairwise comparisons within each condition following a significant condition × time interaction. Black # and ### symbols indicate significant differences between conditions (CP > placebo) at a specific time point following a significant condition × time interaction (*p* < 0.05 and *p* < 0.001, respectively).

MVIC torque (Figure 3a) showed a main effect of time (*F*(3, 126.00) = 267.200, *p* < 0.001), with no condition × time interaction (*F*(3, 126.00) = 0.098, *p* = 0.961) or main effect of condition (*F*(1, 126.00) = 3.332, *p* = 0.070). Values differed across time points (Pre > R5 > Post, R1; all *p <* 0.001), except between Post and R1 (*p =* 0.426).

PRDT (Figure 3b) showed a main effect of time (*F*(3, 21.45) = 40.85, *p* < 0.001), with no interaction (*F*(3, 68.00) = 0.692, *p* = 0.560) or main effect of condition (*F*(1, 17.02) = 0.059, *p* = 0.811). Values differed across time points (Pre > R5 > R1 > Post; all *p <* 0.001).

VA% (Figure 3c) showed a significant condition × time interaction (*F*(3, 102.00) = 2.887, *p* = 0.039). In both CP and placebo conditions, VA% was higher at Pre and R5 than at Post and R1 (CP: *p* ≤ 0.026; placebo: all *p* < 0.001), with no significant difference between Pre and R5 (CP: *p* = 0.384; placebo: *p* = 0.289) and no significant difference between Post and R1 (CP: *p* = 0.437; placebo: *p* = 0.493). Furthermore, VA% at Post (mean difference: 14.74%, 95% confidence interval [CI]: 6.42%–23.06%, *p* < 0.001) and R1 (mean difference: 8.84%, 95% CI: 0.52%–17.17%, *p* = 0.037) were significantly higher in the CP condition than in the placebo condition (Pre: *p* = 0.936; R5: *p* = 0.802).

SDT (Figure 3d) showed a main effect of time (*F*(3, 23.25) = 4.564, *p* = 0.012), with no condition × time interaction (*F*(3, 69.06) = 1.551, *p* = 0.209) or main effect of condition (*F*(1, 17.08) = 3.650, *p* = 0.073). Values at Post (*p* = 0.012) and R1 (*p* = 0.001) were significantly higher than at Pre, and the value at R1 was significantly higher than at R5 (*p* = 0.003).

**FIGURE 4:**
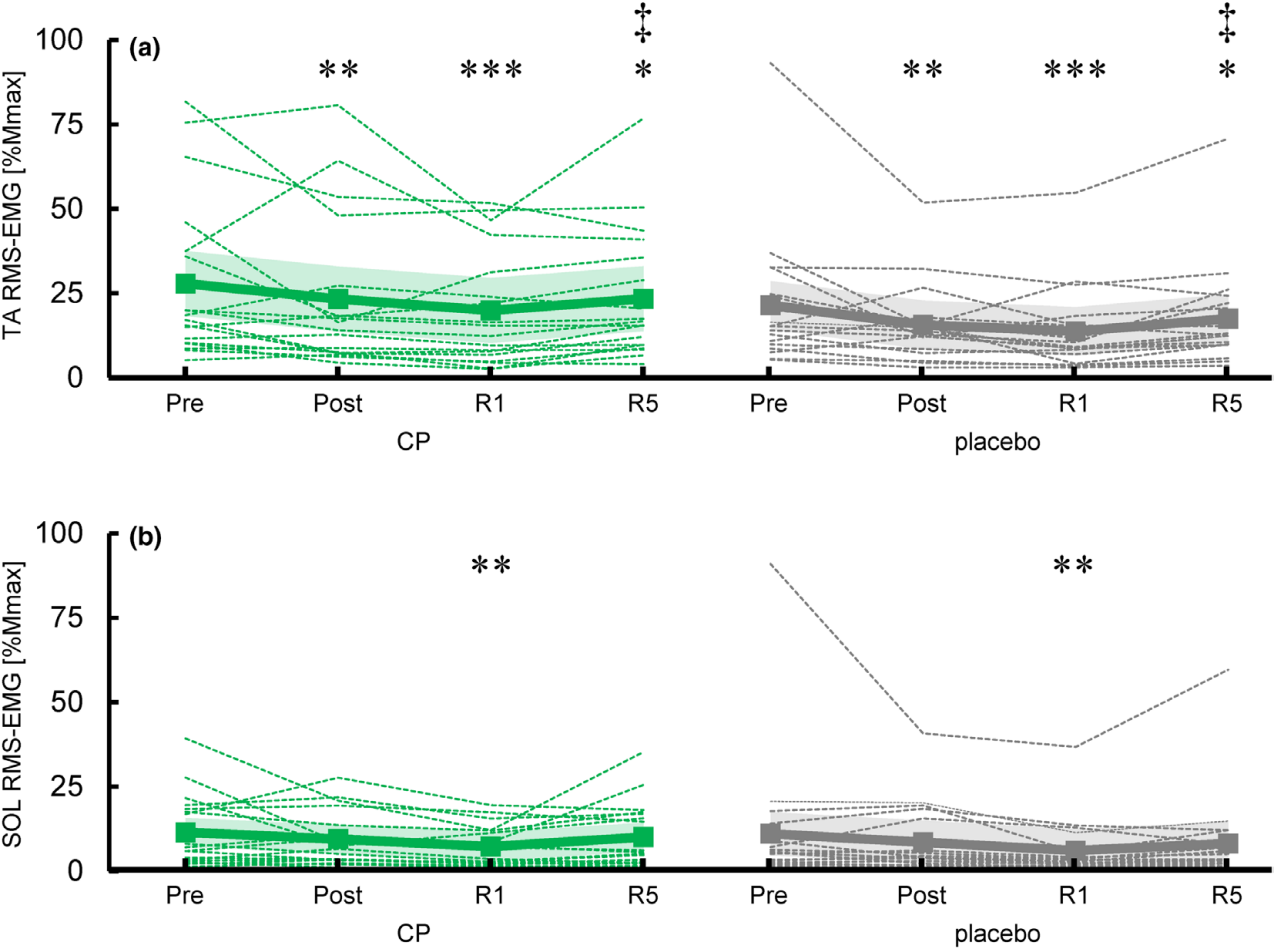
Time course of root mean square electromyographic activity (RMS‐EMG) normalized to the peak‐to‐peak maximal compound muscle action potential amplitude (Mmax) of the tibialis anterior (TA; a) and soleus (SOL; b) muscles during maximal voluntary isometric contraction of the dorsiflexors in the cacao polyphenols (CP; green) and placebo (gray) conditions (*n* = 18). Data are presented as individual participant values (dashed lines) and estimated marginal means (solid lines with squares). Shaded bands represent the 95% confidence intervals derived from the linear mixed model. Measurements were taken at four time points: Before (Pre), immediately after (Post) the fatiguing task, and during recovery at 1 and 5 min post‐task (R1 and R5). The black * (*p* < 0.05), ** (*p* < 0.01), and *** (*p* < 0.001) symbols indicate significant differences from Pre and the black ‡ (*p* < 0.05) symbol indicates a significant difference from R1, based on a significant main effect of time with no significant condition × time interaction.

Normalized RMS‐EMG showed a main effect of time (TA: *F*(3, 102.00) = 9.191, *p* < 0.001; SOL: *F*(3, 102.00) = 4.614, *p* = 0.005), with no interaction or main effect of condition (*p* ≥ 0.280). Post hoc comparisons revealed muscle‐specific differences: for TA (Figure 4a), normalized RMS‐EMG was significantly higher at Pre than at Post, R1, and R5 (*p* ≤ 0.011), and was higher at R5 than at R1 (*p* = 0.026); for the SOL (Figure 4b), a significant difference was observed only between Pre and R1 (Pre > R1; *p* = 0.001).

**FIGURE 5:**
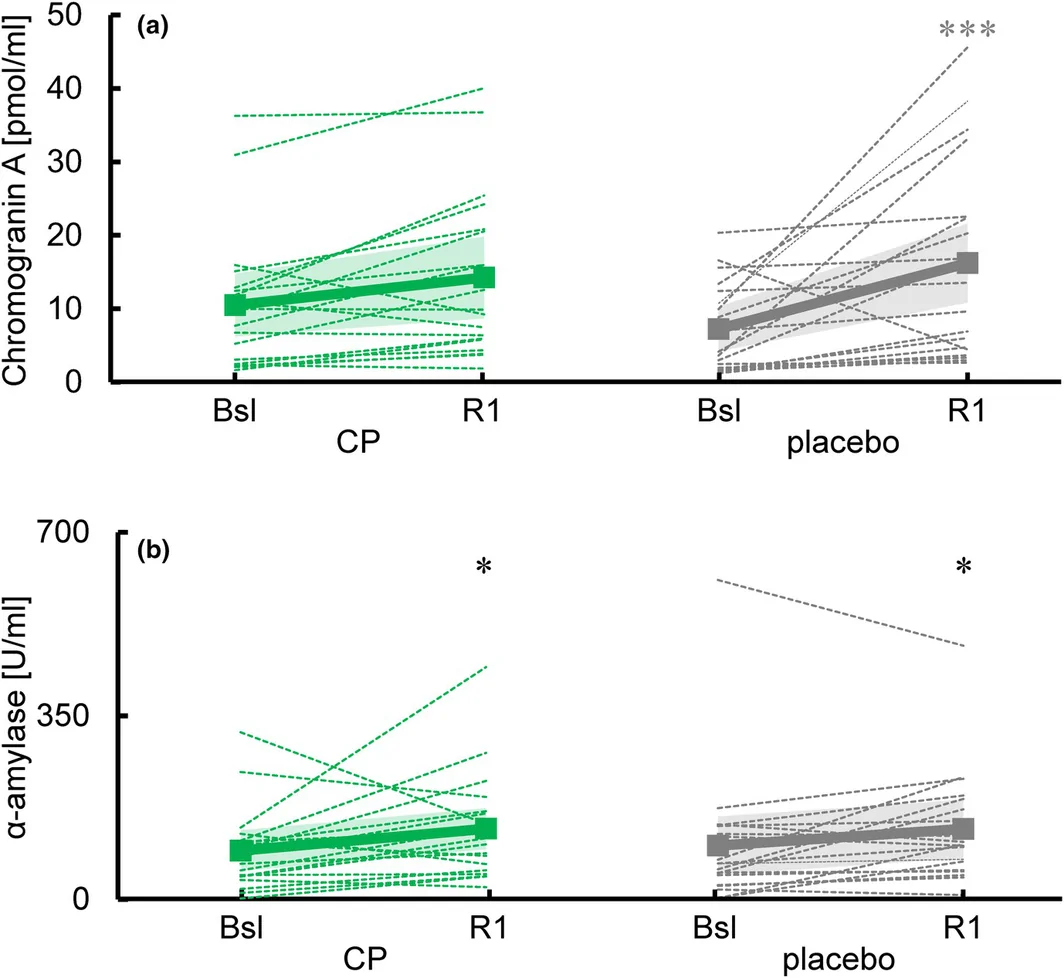
Salivary chromogranin A (a) and α‐amylase (b) before ingestion of the designated food product (Bsl) and during recovery at 1 min post‐task (R1) (*n* = 19) in the cacao polyphenols (CP; green) and placebo (gray) conditions. Data are presented as individual participant values (dashed lines) and estimated marginal means (solid lines with squares). Shaded bands represent the 95% confidence intervals derived from the linear mixed model. The black * (*p* < 0.05) symbol indicates significant differences from Bsl based on a significant main effect of time with no significant condition × time interaction. The gray *** (*p* < 0.001) symbol indicates significant differences from Bsl within the placebo condition following a significant condition × time interaction.

For salivary chromogranin A (Figure 5a), a significant condition × time interaction was observed (*F*(1, 18) = 5.81, *p* = 0.027). Salivary chromogranin A significantly increased from Bsl to R1 only in the placebo condition (*p* < 0.001), whereas no significant change was observed in the CP condition (*p* = 0.083). No significant differences between conditions were observed at either Bsl or R1 (*p* = 0.177–0.417). For α‐amylase (Figure 5b), neither the condition × time interaction (*F*(1, 36) = 0.137, *p* = 0.714) nor the main effect of condition (*F*(1, 18) = 0.045, *p* = 0.834) was significant. However, the main effect of time was significant (*F*(1, 36) = 7.342, *p* = 0.010), with α‐amylase levels being higher at R1 than at Bsl.

## 4 DISCUSSION

The primary findings were that (1) time to task failure did not differ between conditions; (2) among the assessed neuromuscular variables, a significant condition × time interaction was observed only for VA%, with higher values in the CP condition than in the placebo condition at Post and R1; and (3) salivary chromogranin A levels did not significantly increase in the CP condition, unlike in the placebo condition, whereas α‐amylase levels did not differ between conditions. These results partially support our hypotheses.

A single oral dose of CP before the fatiguing task did not improve performance, as assessed by time to task failure. However, LMM analysis showed that VA% was higher in the CP condition than in the placebo condition at Post and R1 (Figure 3c). Because changes in VA% are an established marker of central fatigue (Burnley et al., 2012), these findings indicate that a single CP dose attenuates the decline in central motor drive. Importantly, mechanisms supported by direct measurements should be distinguished from those that remain speculative. The preservation of VA% directly supports the hypothesis that CP modulates central motor drive during and after exercise. Furthermore, the attenuated increase in salivary chromogranin A (Figure 5a) suggests that acute CP ingestion also blunts exercise‐induced autonomic stress responses. In contrast, potential changes in cerebral blood flow, direct brain monoamine kinetics, or localized spinal persistent inward currents were not directly measured in this study and remain speculative. Previous research suggests that acute CP supplementation can enhance cognitive and cerebrovascular function, potentially increasing cerebral oxygenation and vascular reactivity during demanding tasks (Gratton et al., 2020). Additionally, our previous research suggests the possibility that CP modulates the locus coeruleus–noradrenergic network via the gut–brain axis (Fujii et al., 2025). Noradrenaline from the locus coeruleus projects to regions including the prefrontal cortex, hypothalamus, hippocampus, and medulla oblongata, modulating neuronal activity (Breton‐Provencher et al., 2021). Although we did not directly measure central hemodynamics or neurotransmitter kinetics, the observed preservation of VA% may be related to optimized neurovascular coupling (Burma et al., 2025) or to the modulation of persistent inward currents in spinal motoneurons via descending noradrenergic signaling (Heckman et al., 2008). Thus, CP ingestion may trigger a central cascade that helps maintain motor output during and after exhaustive exercise, although direct physiological evidence is needed.

Normalized RMS‐EMG, another indicator of central fatigue (Place et al., 2010), showed no significant CP effect (Figure 4a,b), in contrast to the VA% findings. Normalized RMS‐EMG is susceptible to compensatory motor unit recruitment and rate‐coding changes during fatigue (Farina et al., 2014), which can obscure underlying central deficits. In contrast, superimposed stimulation for VA% bypasses these dynamic compensations to directly quantify central drive failure at the spinal and supraspinal levels (Gandevia, 2001). Thus, this discrepancy is not contradictory; rather, the effect of CP on central motor drive may have been detected more sensitively by VA%.

Furthermore, because VA% is mathematically derived from both SDT (reflecting central drive) and PRDT (reflecting peripheral contractility), changes in VA% could be influenced by alterations in either or both components. In the present study, PRDT showed no significant differences between conditions at any time point (Figure 3b), making differential peripheral contractility an unlikely confounding factor. Regarding SDT (Figure 3d), although a main effect of time was observed, neither the condition × time interaction nor the main effect of condition was statistically significant. This indicates that both conditions exhibited comparable temporal changes. Together, the VA%, PRDT, and SDT findings suggest that CP ingestion may influence central fatigue pathways, although the specific neural adaptations require further verification.

The salivary biomarker findings partially supported the effects of CP on the central nervous system. A significant condition × time interaction was observed for chromogranin A, characterized by a task‐induced increase at R1 in the placebo condition that was effectively blunted in the CP condition (Figure 5a). Conversely, although α‐amylase increased post‐task, no differences were observed between conditions (Figure 5b). Both biomarkers reflect sympathetic activity (Chojnowska et al., 2021), but chromogranin A more directly reflects sympathetic nerve activity, whereas α‐amylase reflects downstream receptor‐mediated secretion and shows a more transient response (Obayashi, 2013). The absence of α‐amylase differences may reflect limited assay sensitivity or the timing of sample collection, despite clear exercise and condition effects observed for chromogranin A. Therefore, the divergence between these two biomarkers is not unexpected. The selective suppression of chromogranin A in the CP condition suggests that pre‐task CP ingestion attenuated excessive sympathetic stress activation during and after the task. This aligns with preserved central motor drive, as indicated by higher VA%. Importantly, changes in PRDT, a direct index of peripheral fatigue (Burnley et al., 2012), did not differ between conditions. This dissociation, characterized by CP affecting specific central and sympathetic indices (VA% and chromogranin A) without altering peripheral contractility or broad systemic indices, suggests that the primary effects of CP are mediated through the central nervous system and its associated autonomic pathways.

However, this greater preservation of central drive did not lead to enhanced overall muscular performance. No differences were observed between conditions in the time course of MVIC torque recovery (Figure 3a) or in time to task failure. These findings suggest that, although CP modulates central nervous system responses to fatigue, its effect on functional performance appears limited under the specific conditions of the 25% MVIC sustained contraction task used here. This may be because, as shown in Figure 3b, PRDT decreased by more than 70% after the fatiguing task, indicating greater‐than‐expected peripheral fatigue. This marked peripheral impairment may have limited the translation of preserved central drive into measurable improvements in functional performance. Thus, while the higher %VA observed following CP ingestion provides preliminary evidence that CP may influence central drive, the absence of corresponding CP treatment effects on overall muscular performance, as well as on PRDT, SDT, and normalized RMS‐EMG, indicates that further confirmation of centrally mediated effects of CP is required.

This study has several limitations. First, we used a sustained isometric contraction at 25% MVIC. Although this intensity was selected to induce central fatigue (Place et al., 2009), as noted, the substantial peripheral impairment may have limited the functional expression of central benefits from CP. Future studies should examine whether CP provides functional benefits during higher‐intensity or intermittent tasks, or at lower intensities where peripheral homeostasis is better maintained and central drive plays a larger role. Second, salivary biomarkers were assessed at a single post‐task time point (R1). Although this time point was appropriate for capturing acute responses, more frequent sampling during recovery may better characterize the time course of stress responses and the duration of CP's effect on chromogranin A. Third, direct measurement of central parameters was beyond the scope of this study; mechanisms such as cerebral blood flow or brain neurotransmitter kinetics remain speculative and warrant future direct neuroimaging or neurochemical investigation. Fourth, whether these acute effects are specific to CP or apply to dietary polyphenols more broadly remains to be determined through comparative studies using different polyphenol sources. Fifth, the study included only healthy young male adults to reduce variability related to sex‐specific hormonal variation; thus, generalizability to females remains unclear. Finally, an optimal timing of ingestion and a dose–response relationship were not assessed in the present study, which should be considered in future experiments.

Despite these limitations, the present findings suggest that acute CP ingestion may attenuate central fatigue and physiological stress, as evidenced by the preservation of VA% and suppression of salivary chromogranin A. These results raise the possibility that CP may influence the locus coeruleus–noradrenergic network via the gut–brain axis, thereby maintaining central motor drive while mitigating excessive sympathetic stress activation during and after exhaustive tasks. However, the lack of improvement in functional performance suggests that the ergogenic effects of CP may be intensity‐dependent. In this study, the substantial peripheral fatigue indicated by the marked decrease in PRDT may have attenuated the functional expression of preserved neural drive. Therefore, CP may be more beneficial in athletic settings in which peripheral metabolic disturbances are minimized and central drive or autonomic stress modulation serves as the primary limiting factor, such as prolonged very‐low‐intensity activities or tasks requiring high cognitive engagement. Furthermore, optimizing intake protocols may be necessary to translate these central effects into functional gains. Future research should investigate the effects of chronic dosing and administration timing, such as pre‐versus post‐training intake, while accounting for sex‐specific hormonal and autonomic variations to inform strategies for diverse athletic populations.

## 5 CONCLUSION

This study indicates that a single oral dose of CP attenuates the decline in central motor drive and suppresses the acute autonomic stress response following a sustained submaximal fatiguing task at 25% MVIC of the dorsiflexors, as indicated by higher VA% at Post and R1 and suppressed salivary chromogranin A increase at R1. Although these central and autonomic effects did not improve time to task failure or MVIC torque recovery, the findings provide preliminary evidence that acute CP ingestion modulates central nervous system and sympathetic responses to strenuous exercise.

## AUTHOR CONTRIBUTIONS

**Ikumi Morikawa:** Conceptualization; data curation; formal analysis; investigation; methodology; validation; visualization. **Ashiyu Ono:** Data curation; formal analysis; investigation; validation; visualization. **Yuta Osada:** Investigation. **Yasuyuki Fujii:** Validation. **Midori Natsume:** Validation. **Naomi Osakabe:** Conceptualization; funding acquisition; methodology; project administration; resources; supervision; validation. **Ryota Akagi:** Conceptualization; data curation; formal analysis; funding acquisition; methodology; project administration; resources; supervision; validation; visualization.

## FUNDING INFORMATION

This study was supported by research grants from Meiji Co., Ltd. and Meiji Holdings Co., Ltd. (to N.O. and R.A., respectively).

## CONFLICT OF INTEREST STATEMENT

This study was funded by Meiji Co., Ltd. and Meiji Holdings Co., Ltd. M.N. is an employee of Meiji Co., Ltd. and Meiji Holdings Co., Ltd. and holds shares in the company but holds no relevant patents. Nevertheless, the study was conducted as a double‐blind randomized crossover trial. M.N. was responsible for providing the test and placebo samples but was not involved in direct data collection, blinded data analysis, or the decision to publish the results. Apart from these interests, the authors declare no conflicts of interest.

## Data Availability

The datasets generated and/or analyzed in this study are available from the corresponding author upon reasonable request.
